# Endoscopic detection of near-infrared autofluorescence in the parathyroid glands: a case report of two patients

**DOI:** 10.1097/MS9.0000000000005247

**Published:** 2026-06-19

**Authors:** Ryuhei Okada, Kazuchika Ohno, Iichiroh Onishi, Takahiro Asakage

**Affiliations:** a Department of Head and Neck Surgery, Institute of Science Tokyo, Tokyo, Japan; b Department of Pathology, Institute of Science Tokyo, Tokyo, Japan

**Keywords:** case report, fluorescence imaging, parathyroid, VANS, video-assisted neck surgery

## Abstract

**Introduction and importance:**

Preservation of the parathyroid glands during thyroid surgery is crucial to prevent postoperative hypocalcemia. Parathyroid glands exhibit intrinsic autofluorescence with an emission peak around 820 nm, and near-infrared imaging has been applied for their intraoperative identification. However, its feasibility in endoscopic thyroid surgery remains unclear, particularly when using a small-diameter endoscope. Here, we report two cases in which parathyroid autofluorescence was successfully visualized during endoscopic surgery using a 5-mm endoscope.

**Case presentation:**

The first patient was a 75-year-old woman with breast cancer who was referred to us with a right thyroid nodule detected on preoperative positron emission tomography/computed tomography. Fine-needle aspiration cytology suggested papillary carcinoma, and as the nodule gradually enlarged, we performed a right thyroid lobectomy after 7 years using video-assisted neck surgery (VANS) with a 5-mm endoscope. A parathyroid gland on the posterior thyroid surface showed homogeneous and strong autofluorescence. The second patient was a 53-year-old woman with primary hyperparathyroidism who presented with hypercalcemia and elevated intact parathormone levels. We performed a right parathyroidectomy using VANS with the same endoscope. A parathyroid tumor showing heterogeneous and relatively weak autofluorescence was identified on the posterior aspect of the right thyroid lobe.

**Clinical discussion:**

In both cases, near-infrared imaging revealed parathyroid autofluorescence during VANS. Adequate display settings, including enhanced brightness and contrast, appear to be important for clear visualization.

**Conclusion:**

Parathyroid autofluorescence can be visualized even during endoscopic thyroid surgery with a 5-mm endoscope and may contribute to safer procedures.

## Introduction

It is important to preserve the parathyroid glands during thyroid surgery. A decline in parathyroid function may result in hypocalcemia, and oral supplementation with vitamin D and calcium is required; however, prolonged administration of calcium and vitamin D could lead to complications, including renal dysfunction[1]. Although it would be preferable to locate the parathyroid glands during surgery by gross inspection, their identification is often challenging by visual observation alone. Parathyroid glands are considered to possess intrinsic autofluorescence in the near-infrared range, with an emission peak at an 820-nm wavelength[2], and the usefulness of intraoperative fluorescence imaging has been reported^[^3,4^]^. In addition to conventional visual imaging, probe-type devices employing fluorescence imaging have also been developed in recent years[5]. In cases of hyperparathyroidism, accurate identification of parathyroid adenomas is also of critical importance. Although parathyroid adenomas have been reported to exhibit weaker or heterogeneous autofluorescence compared with normal parathyroid glands^[^6,7^]^, fluorescence imaging may aid in their identification.

HIGHLIGHTS

- Parathyroid autofluorescence was successfully visualized during video-assisted neck surgery.
- Parathyroid fluorescence was detectable even with a small-diameter (5-mm) endoscope.
- Normal parathyroid glands may exhibit stronger and more homogeneous autofluorescence than adenomas.
- Visualization of autofluorescence may depend on the performance of the display system.

Traditionally, surgeries for the thyroid and parathyroid glands have been performed through a cervical incision. However, because a cervical scar may reduce aesthetic patient satisfaction, remote-access approaches for thyroid and parathyroid surgery have been developed. While various remote-access techniques have been reported[8], in Japan, the video-assisted neck surgery (VANS) approach via a subclavicular incision is most commonly employed^[^9,10^]^. The VANS approach can be considered a relatively straightforward technique, as the distance from the incision to the thyroid is comparatively short. The ability to perform the procedure in a gasless manner is also an advantage, as it reduces the risk of potential complications. Critical complications, such as recurrent laryngeal nerve palsy, are reported to occur at rates comparable to those in conventional cervical incisions[11]; therefore, when patient satisfaction is also taken into account, this method can be considered a valuable surgical approach. Nevertheless, to date, reports on the observation of parathyroid autofluorescence during endoscopic surgery are limited, and most of the previous studies have used a 10-mm endoscope^[^12,13^]^. It remains unclear whether parathyroid autofluorescence can be detected with endoscopes smaller than this. Herein, we describe our experience in capturing parathyroid autofluorescence with a fluorescence endoscope during VANS with a 5-mm endoscope.

This case report has been reported in line with the SCARE 2025 checklist[14].

## Device setting

**Figure 1.**
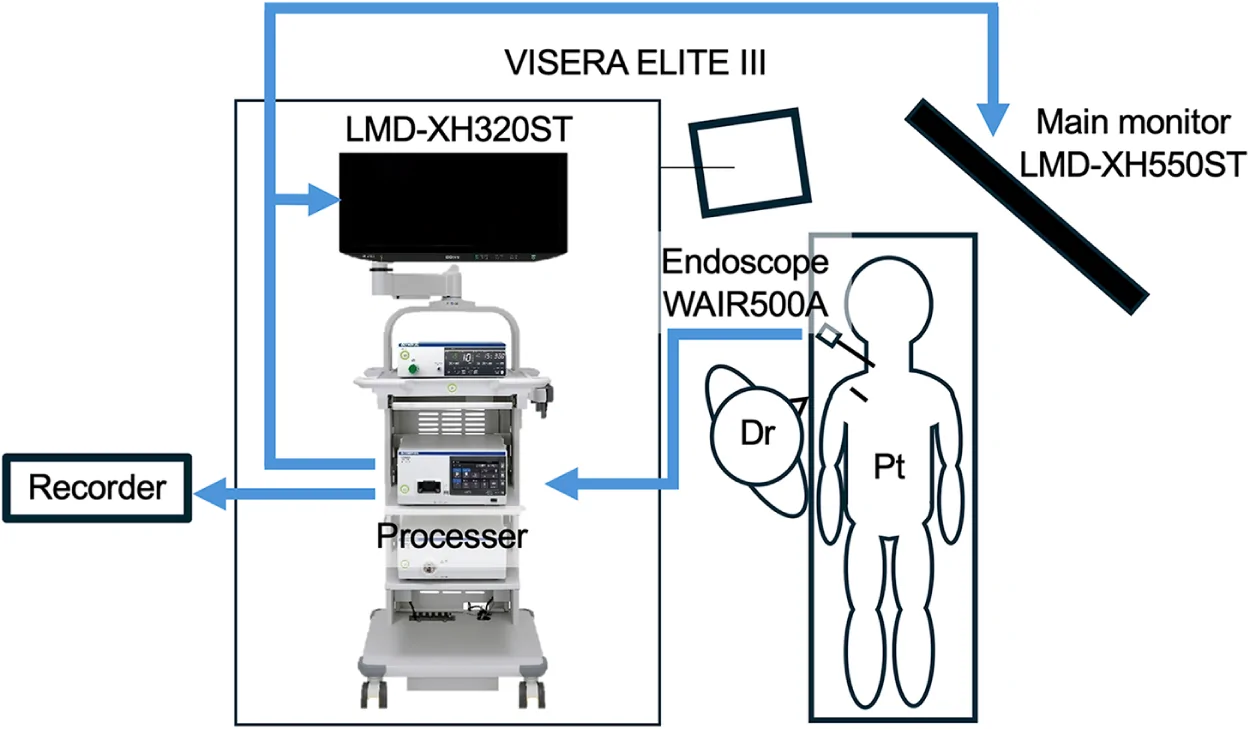
Device settings. Typical device settings for right-sided surgery are shown. A 55-inch display was used as the main display. For fluorescence imaging, a fluorescence telescope (WAIR500A; 5.4 mm, 0 degree) was used with the VISERA ELITE III platform (Courtesy of Olympus Marketing, Inc.). Images were sent to the 4K 55-inch display (LMD-XH550ST), which provides high brightness and high contrast. The same images could be observed on the subdisplay (LMD-XH320ST). The recorder was connected directly to the ELITE III processor without using the display. Dr., doctor; Pt., patient.

In the present cases, we used a VISERA ELITE III platform (Olympus, Japan) with the WAIR500A (5.4 mm, 0 degree; Olympus, Japan), which is equipped with a near-infrared mode (Fig. 1). A 55-inch 4K display (LMD-XH550ST; SONY, Japan) was employed as the main display. This display is characterized by high brightness and the ability to enhance contrast. During near-infrared imaging, all lights in the operating room were turned off, the tip of the endoscope was placed as close as possible to the target, and the brightness was set to +8. The recorder was connected directly to the ELITE III processor without using the display.

## Case presentation

### Case 1

**Figure 2.**
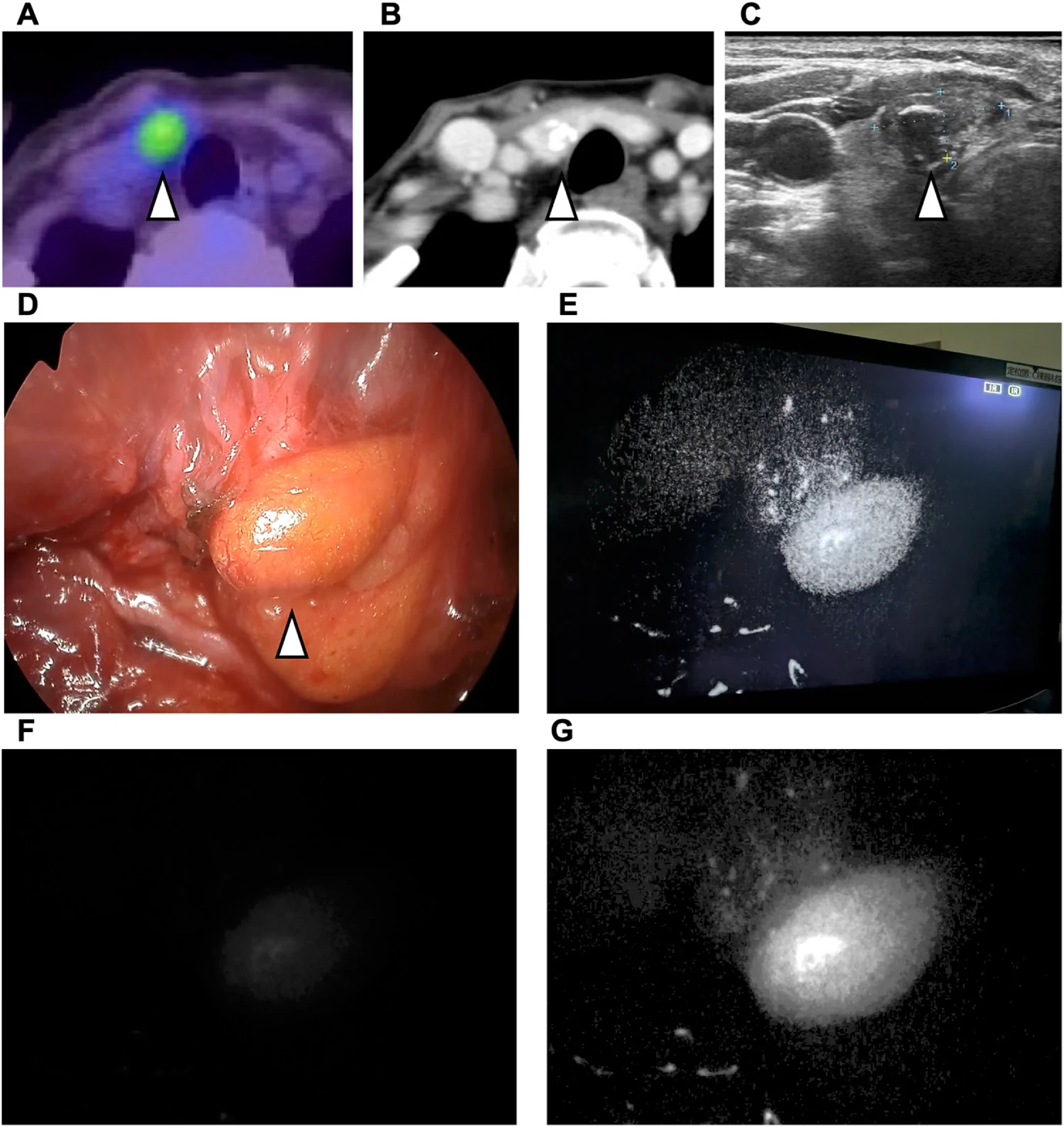
Case 1 (A) A nodule in the right thyroid lobe was detected on preoperative PET/CT performed for breast cancer. (B) Preoperative CT image: A calcified nodule was observed in the right lobe of the thyroid; however, no obvious metastases were detected in the cervical lymph nodes or lungs. (C) Preoperative US image: A 14-mm nodule with calcification was shown in the right lobe of the thyroid. (D) A parathyroid gland adherent to the posterior aspect of the right thyroid lobe was identified. (E) On the display screen, a homogeneous and strong autofluorescence was observed. (F) On the recording obtained from direct connection to the ELITE III system, autofluorescence could not be clearly observed. (G) When the brightness of the recorded images was increased, the fluorescence could be distinguished.

A 75-year-old Asian woman with breast cancer was referred to our department after a preoperative positron emission tomography/computed tomography (PET/CT) revealed abnormal uptake in the right lobe of the thyroid gland (Fig. 2A). A nodule measuring approximately 1 cm was identified in the right lobe of the thyroid, and fine-needle aspiration cytology suggested papillary carcinoma. The patient first underwent breast cancer resection and was subsequently treated with anastrozole, along with a statin for dyslipidemia and eldecalcitol for osteoporosis. The thyroid lesion was carefully followed up during this period; however, as it gradually increased in size over 7 years, surgical intervention was eventually indicated. The detailed timeline is shown in Supplemental Digital Content Figure S1, available at: http://links.lww.com/MS9/B261. Preoperative CT revealed a calcified nodule in the right lobe of the thyroid, but no definite evidence of metastasis (Fig. 2B). Ultrasonography also revealed a 14-mm nodule with calcification in the right lobe of the thyroid (Fig. 2C). Based on the findings, the lesion was diagnosed as papillary thyroid carcinoma (T1bN0M0), and we scheduled a right lobectomy of the thyroid. As the patient expressed her preference for the endoscopic approach, we performed VANS. The procedure was performed under general anesthesia with the patient in the supine position. A 3-cm skin incision was made 7 cm lateral to the midline below the clavicle, and a gasless technique was employed by elevating the skin with a retractor[15]. A 5-mm camera port was additionally created in the anterior neck. Intraoperative recurrent laryngeal nerve monitoring was performed. When the right thyroid lobe was medially rotated, a mass suspected to be a parathyroid gland was identified on the posterior surface of the thyroid (Fig. 2D). A homogeneous and intense autofluorescence was distinctly observed on the display with enhanced brightness and contrast (Fig. 2E). The mass was preserved, and a right thyroid lobectomy was performed. A portion of the mass suspected as a parathyroid gland was submitted for histopathological examination, which confirmed it as parathyroid tissue (Supplemental Digital Content Figure S2, available at: http://links.lww.com/MS9/B261). Upon reviewing the original recorded video, which was not displayed on the display with enhanced brightness and contrast, visualization of parathyroid autofluorescence was difficult (Fig. 2F). However, by digitally adjusting the brightness, we were able to confirm the autofluorescence of the parathyroid glands (Fig. 2G). The operative time was 183 minutes, and blood loss was minimal. She was discharged on postoperative day 3. The surgical wound was protected with tape for one week. No perioperative complications were observed, and no postoperative hypocalcemia occurred. No recurrence of thyroid cancer was observed at 7 months postoperatively.

### Case 2

**Figure 3.**
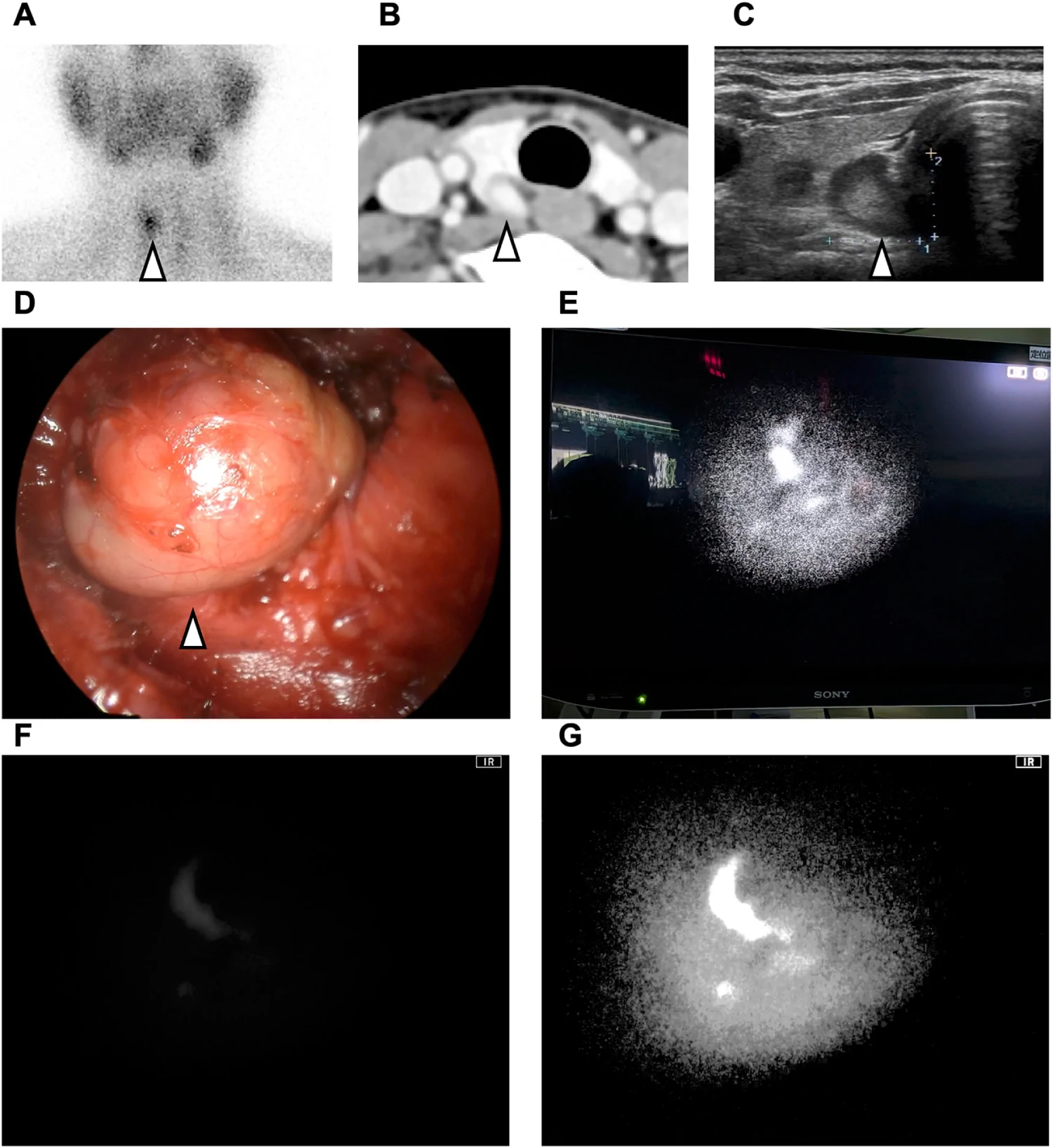
Case 2 (A) MIBI scintigraphic imaging revealed accumulation at the level of the right thyroid lobe. (B) Preoperative CT image: A 1-cm nodule was identified posterior to the right lobe of the thyroid. (C) Preoperative US image: A 9.8-mm nodule was identified posterior to the right lobe of the thyroid. (D) A parathyroid tumor was identified on the posterior aspect of the right thyroid lobe. (E) On the display, heterogeneous and relatively weak fluorescence was observed. (F) On the recording obtained from a direct connection to the ELITE III processor, autofluorescence could not be clearly observed. (G) When the brightness of the recorded images was increased, the fluorescence could be distinguished.

A 53-year-old Asian woman presented with elevated serum calcium (10.9 mg/dL) and intact parathyroid hormone (118 pg/mL) levels. She had a history of dyslipidemia and had been treated with a statin. There was no family history of thyroid or parathyroid disease. MIBI scintigraphy revealed focal uptake corresponding to the right thyroid lobe level, and she was referred to our department with suspected primary hyperparathyroidism caused by a parathyroid tumor (Fig. 3A). Bone densitometry revealed a marked reduction in bone density, with a lumbar spine T-score of −2.3 and Z-score of −1.4. Preoperative CT revealed an enhancing mass located on the dorsal aspect of the right thyroid lobe (Fig. 3B). Ultrasonography also revealed a hypoechoic mass measuring approximately 1 cm in the same region, which was not stiff on elastography (Fig. 3C). A non-stiff nodule was identified at a site corresponding to MIBI uptake, and, given its posterior location relative to the thyroid gland, a parathyroid adenoma was suspected rather than lymph node metastasis or a thyroid nodule. We made a diagnosis of primary hyperparathyroidism, suspected to be caused by a right parathyroid adenoma, and planned surgical intervention. The detailed timeline and laboratory data are presented in Supplemental Digital Content Figure S3, available at: http://links.lww.com/MS9/B261. As the patient expressed a preference for an endoscopic approach, we opted for VANS. The procedure was performed in the same manner as in Case 1. As dissection proceeded along the posterior aspect of the thyroid, a yellowish mass was identified (Fig. 3D). Autofluorescence of the mass was observable on the display capable of enhanced contrast, although the fluorescence was heterogeneous and relatively weak (Fig. 3E). The mass was carefully dissected free from the surrounding tissue and excised. Intraoperative histopathological examination confirmed that the lesion contained parathyroid tissue. The operative time was 231 minutes, and blood loss was minimal. On histopathological examination, the lesion was diagnosed as a parathyroid adenoma, with little intervening adipose tissue and focal hemorrhage (Supplemental Digital Content Figure S4, available at: http://links.lww.com/MS9/B261). Postoperatively, the serum calcium and intact PTH levels decreased promptly. No calcium supplementation was administered, and no hypocalcemia occurred. In this case as well, a review of the recorded video revealed that parathyroid autofluorescence was difficult to visualize in the original footage (Fig. 3F), but became observable after the brightness setting was increased (Fig. 3G). No abnormalities in serum calcium or intact PTH levels were observed during the first postoperative month.

## Discussion

In these two cases, we employed a novel 5-mm endoscopic system and successfully visualized near-infrared autofluorescence of the parathyroid glands in both a normal gland and an adenoma. Intraoperatively, parathyroid autofluorescence could be observed on the display, which was set at an increased brightness and contrast level. However, in the original images not processed through the display, autofluorescence was difficult to discern. Nevertheless, by digitally increasing the brightness of the original images, autofluorescence could be identified, suggesting that observation on a display with enhanced brightness and contrast settings is of particular importance. With current technology, the detection of autofluorescence largely depends on the performance of the display. Because the surgical field in VANS procedures is narrower than that in conventional cervical incisions, observation of parathyroid autofluorescence is considered highly useful. In our cases, we experimented with various conditions and found that clear visualization of autofluorescence could be achieved by turning off the room lights, positioning the camera lens as close as possible to the target, and setting the brightness on the system to the maximum level (+8). In the future, advances in endoscopes and related devices may enable easier observation of autofluorescence. It has been reported that normal parathyroid glands exhibit more intense and homogeneous autofluorescence compared with adenomas, which may be explained by the histological features of functional parathyroid adenomas, including increased cellularity, patchy areas of fibrosis, oxyphilic cell clusters, and areas of hematoma^[^6,7^]^, and our findings in the present cases were consistent with this previous report. However, further accumulation of cases will be required to draw definitive conclusions.

In recent years, the introduction of artificial intelligence (AI) technology into diagnostic imaging has been remarkable. Reports have also demonstrated the usefulness of AI in the assessment of parathyroid autofluorescence. According to Yu *et al*, an AI model for parathyroid autofluorescence imaging achieved a recognition rate of 82.4% in external validation, which was significantly superior to that achieved by junior surgeons[16]. While in the present study we confirmed autofluorescence with our own eyes through the display, in the future, the development of systems capable of AI-based discrimination might be desirable.

Besides autofluorescence, indocyanine green (ICG) fluorescence imaging is another known technique for identifying the parathyroid glands. This involves the intravenous administration of ICG during surgery, followed by near-infrared imaging, which facilitates both the identification of the parathyroid glands and the evaluation of their blood supply[17]. According to Tae *et al*, the use of ICG imaging in robotic thyroidectomy did not enhance parathyroid gland identification or decrease the rate of hypocalcemia; however, it did allow the prediction of hypocalcemia development[18]. In the present cases, we did not perform ICG-based imaging; however, the current endoscopic system is also capable of visualizing ICG, and we plan to investigate its utility in the future.

This study has several limitations. First, our observations are based on only two cases, and therefore, the generalizability of the findings is limited. Second, the detection of parathyroid autofluorescence may be affected by false-positive or false-negative findings. In addition, visualization of autofluorescence partly depends on subjective perception by the surgeon and the settings of the imaging display. Finally, the feasibility of this technique may depend on the availability and performance of specific imaging equipment, which may not be widely accessible in all surgical settings. Further studies with larger numbers of patients are required to validate these findings.

## Conclusion

Herein, we report the successful observation of parathyroid autofluorescence during surgery using a novel 5-mm endoscopic system, both in a normal gland and in an adenoma. This technique has the potential to become a standard approach in future endoscopic thyroid and parathyroid surgery.

## Data Availability

No datasets were generated or analyzed during the current study. Data sharing is not applicable to this article because it is based on two patient case reports.
